# Variations on a Theme: Antennal Lobe Architecture across Coleoptera

**DOI:** 10.1371/journal.pone.0166253

**Published:** 2016-12-14

**Authors:** Martin Kollmann, Rovenna Schmidt, Carsten M. Heuer, Joachim Schachtner

**Affiliations:** 1 Department of Biology—Animal Physiology, Philipps-University Marburg, Marburg, Germany; 2 Institute of Veterinary Anatomy, Histology and Embryology, Justus-Liebig University Gießen, Gießen, Germany; 3 Fraunhofer-Institut für Naturwissenschaftlich-Technische Trendanalysen INT, Euskirchen, Germany; University of Mississippi, UNITED STATES

## Abstract

Beetles comprise about 400,000 described species, nearly one third of all known animal species. The enormous success of the order Coleoptera is reflected by a rich diversity of lifestyles, behaviors, morphological, and physiological adaptions. All these evolutionary adaptions that have been driven by a variety of parameters over the last about 300 million years, make the Coleoptera an ideal field to study the evolution of the brain on the interface between the basic bauplan of the insect brain and the adaptions that occurred. In the current study we concentrated on the paired antennal lobes (AL), the part of the brain that is typically responsible for the first processing of olfactory information collected from olfactory sensilla on antenna and mouthparts. We analyzed 63 beetle species from 22 different families and thus provide an extensive comparison of principal neuroarchitecture of the AL. On the examined anatomical level, we found a broad diversity including AL containing a wide range of glomeruli numbers reaching from 50 to 150 glomeruli and several species with numerous small glomeruli, resembling the microglomerular design described in acridid grasshoppers and diving beetles, and substructures within the glomeruli that have to date only been described for the small hive beetle, *Aethina tumida*. A first comparison of the various anatomical features of the AL with available descriptions of lifestyle and behaviors did so far not reveal useful correlations. In summary, the current study provides a solid basis for further studies to unravel mechanisms that are basic to evolutionary adaptions of the insect olfactory system.

## Introduction

Beetles first appeared in the early Permian (around 270–300 million years ago) [1–3]. Their evolutionary success appears to have been sparked by an initial burst of speciation and consolidated through high diversification and low extinction rates throughout history [4]. This has been attributed to their effective adaptation to geological and climatic changes [5] and a coleopteran co-evolution with mammals [6] and angiosperms [7].

Today, Coleoptera is the most species-rich metazoan order. With about 400,000 described species, beetles represent approximately 30% of all known animal species [2,8–10]. Based on this enormous species richness, Coleoptera display a vast diversity of lifestyles and behaviors, inhabiting all biomes but the marine environment and comprising, inter alia, nocturnal and diurnal species, mutualistic and parasitic symbionts, generalists and specialists, carnivorous, herbivorous, detritivorous and coprophagous taxa [11].

This huge diversity is mirrored by numerous physiological and morphological adaptations. We here seek to explore whether the diversity is also reflected by neuroanatomical adaptions in the central nervous system. Beetles provide an excellent opportunity to explore the extent of such adaptions within a single insect order. Since olfaction plays a prominent role in the life history of insects (finding food, hosts, mates etc.; [12–17], we focused our investigation on the primary olfactory neuropil, the paired antennal lobes (ALs).

In insects, olfactory information is detected by olfactory sensory neurons (OSNs) housed in olfactory sensilla on the antennae and the labial and/or maxillary palps of the mouthparts [18–20]. Via the antennal nerves (ANs), olfactory input from the antennae is passed on to the ALs, the first integration centers for olfactory information. Typically, the ALs comprise spherical subcompartments, the olfactory glomeruli [20,21] and also typically, all OSNs expressing the same type of olfactory receptor (OR) converge onto the same glomerulus [22]. The number of glomeruli can vary among different species, ranging from about 40 to sometimes several hundred [21,23–25]. Within the ALs, the olfactory information is processed by a complex network of neurons, including OSNs, local interneurons (LNs), projection neurons (PNs), and centrifugal neurons (CNs) [21]. The olfactory representation within the ALs is shaped by the neuronal network and by a variety of neuroactive substances, most notably the inhibitory transmitter gamma amino-butyric acid (GABA), the excitatory transmitter acetylcholine [26–32] but also biogenic amines, neuropeptides like e.g. Tachykinin-related peptides (TKRP), and gaseous signaling molecules [21, 33–35]. The PNs forward the processed olfactory information via antennal lobe tracts (ALTs) to higher brain centers (in particular the mushroom bodies [MBs] and the lateral horns [LHs] [21,36]).

Despite their diversity and species richness, as well as their preeminent ecological and economic importance [2,8], a comprehensive and comparative analysis of the coleopteran olfactory system has not been conducted to date. Detailed information on the ALs of Coleoptera is scarce [21]—only the ALs of the scarab beetle *Holotrichia diomphalia* [37], of the red flour beetle *Tribolium castaneum* [34,38,39], and of the small hive beetle *Aethina tumida* [40] have been investigated in greater detail. Exhibiting 60–90 spherical glomeruli, the ALs in these species conform to the basic bauplan of a typical insect AL [21]. However, for some beetle species, atypical AL anatomies have been reported. The ALs of Dytiscinae (diving beetles) have been described as non-glomerular [41–43] and ALs seem to be missing altogether in aquatic Gyrinidae (whirligig beetles)–possibly representing a loss-of-function and indicating anosmia in these animals [43,44]. However, a recent study found numerous small glomeruli within Dytiscinae [45]. Recent investigations in *A*. *tumida*, using antibodies against TKRP, a neuropeptide known to modulate olfactory sensitivity and locomotor activity in the fruit fly *Drosophila melanogaster* [46–49] and the cockroach *Periplaneta americana* [50], revealed hitherto undescribed substructures within the olfactory glomeruli [40].

In the current study, we used the anti-TKRP antiserum in combination with anti-synapsin antibody staining and phalloidin staining to investigate whether the glomerular substructures described for *A*. *tumida* can also be found in other Coleoptera. We investigated the AL of 63 beetle species from 22 different families, thus providing the most exhaustive dataset on AL neuroarchitecture within an insect order to date. Glomeruli numbers were obtained for 32 of the examined beetle species, reaching from 50 to 150 glomeruli (with 80 to 120 glomeruli in the majority of animals) and revealing much more diversity than would be expected from existing studies within Coleoptera [34,37–40]. The observed neuroanatomical diversity of coleopteran AL organization also includes several species with numerous small glomeruli (comparable to the situation in acridid grasshoppers and diving beetles) and AL substructures recently described for the small hive beetle, *Aethina tumida* [40].

## Results

### General architecture and number of glomeruli within the coleopteran antennal lobes

**Fig 1:**
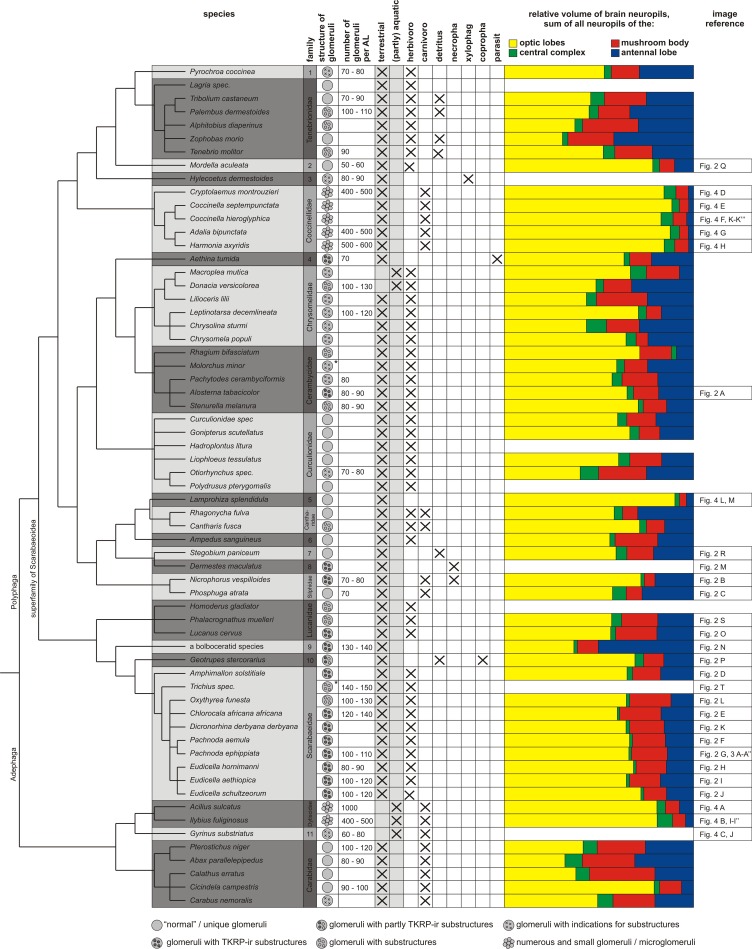
Phylogenetic tree of the investigated Coleopteran species. The phylogenetic tree providing information on the design of the antennal lobes, lifestyles (information on habitat and major nutrition) and relative neuropil volumes. Families in which only a single species was examined are: 1 = Pyrochroidae; 2 = Mordellidae; 3 = Lymexylidae; 4 = Nitidulidae; 5 = Lampyridae; 6 = Elateridae; 7 = Ptinidae; 8 = Dermestidae; 9 = Bolboceratidae; 10 = Geotrupidae; 11 = Gyrinidae. Icons to the right of the family names show whether AL substructures could be observed and whether these are immunoreactive to tachykinin-related peptide (TKRP) or if a microglomerular organization could be observed (see legend at the bottom; *: no immunostainings against TKRP are available). Data on lifestyle of the animals extracted from: [98–102].

We obtained numbers of olfactory glomeruli in 32 coleopteran species (Fig 1). With regard to their general neuroanatomical makeup, the ALs could be categorized into two groups: 1) ALs containing 50–150 more or less spherical or oval shaped glomeruli of a regular size, typically arranged around a central coarse neuropil, comparable to the conditions found in the majority of insects (e.g. in Diptera, Hymenoptera, or Lepidoptera, [21]). In the majority of the examined beetles, the number of glomeruli per AL ranges from 80 to 120 glomeruli. 2) ALs comprising approximately 400–1,000 small glomeruli, comparable to the microglomeruli of locusts and other Acrididae [21,51]. Interestingly, within Coleoptera, such microglomeruli are only observed within two families that are not closely related to each other (Coccinellidae and Dytiscidae; see below). In general, the number of glomeruli does not vary much within families, with the exception of Dytiscidae (one species with about 1,000 and one with about 400–500 glomeruli).

### TKRP-immunoreactive substructures in antennal lobe glomeruli

Among the 63 investigated beetle species, the olfactory glomeruli of almost 25% exhibited glomerular substructures that labeled with the TKRP antibody similar to those described in *A*. *tumida* [40]. In addition to the Nitidulid *A*. *tumida*, such TKRP-immunoreactive (TKRP-ir) glomerular substructures were observed in representatives of six different families (Cerambycidae, Dermestidae, Silphidae, Lucanidae, Bolboceratidae, and Scarabaeidae). However, careful in-group comparisons in four families revealed that TKRP-ir substructures cannot per se be regarded as characteristic of a distinct family.

**Fig 2:**
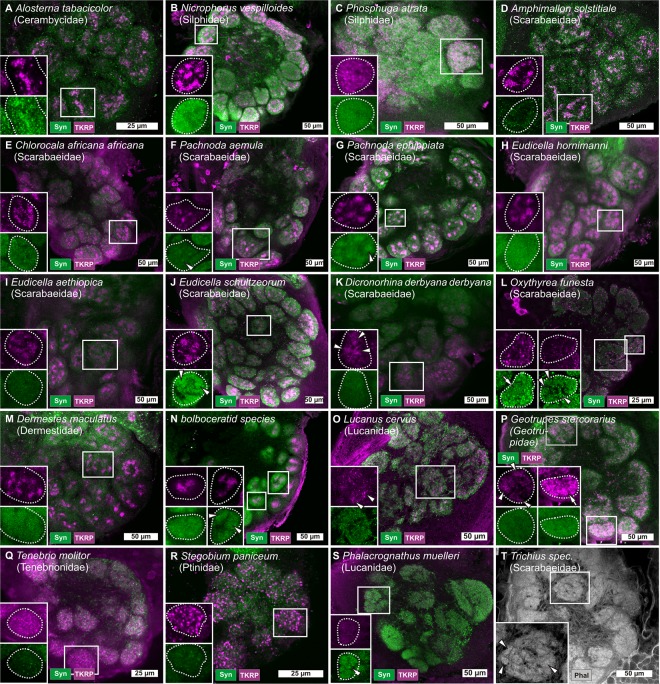
Antennal lobes of Coleoptera. Coleopteran antennal lobes (ALs) labeled with various markers (immunostainings against synapsin [Syn, green] and Tachykinin-related peptide [TKRP, magenta] as well as phalloidin labeling [Phal, grey]). Boxes in the lower left corner show details of single glomeruli, marked in the overview images. Arrowheads show glomerular substructures (F, G, J—L, N—P, S, and T), arrows show homogenously stained glomeruli (L, P).

For example, within Cerambycidae (longhorn beetles), only *A*. *tabacicolor* exhibits TKRP-ir substructures (Fig 2A). In the Cerambycidae *P*. *cerambyciformis*, *S*. *melanura*, and *R*. *bifasciatum* TKRP-ir fibers/areas can be observed in various regions of the brain (primarily in the protocerebrum) but in the ALs, marked TKRP-ir stainings were absent. In the Silphidae (burying beetles) *N*. *vespilloides* and *P*. *atrata* were investigated. While the former possesses well defined TKRP-ir substructures within its glomeruli (Fig 2B), the AL of *P*. *atrata* exhibit a homogeneous TKRP-ir staining pattern that does not indicate such structuring (Fig 2C). The family in which we identified the most species exhibiting TKRP-ir substructures are the Scarabaeidae. Within this family, seven of the investigated species display well-defined TKRP-ir substructures within their glomeruli (*A*. *solstitiale* [Fig 2D], *C*. *africana africana* [Fig 2E], *P*. *aemula* [Fig 2F], *P*. *ephippiata* [Fig 2G], *E*. *hornimanni* [Fig 2H], *E*. *aethiopica* [Fig 2I], and *E*. *schultzeorum* [Fig 2J]), while one Scarabaeidae species (*D*. *derbyana derbyana* [Fig 2K]) exhibits only weakly demarcated TKRP-ir substructures. Another Scarabaeidea species (*O*. *funesta* [Fig 2L]) possesses a granular TKRP-ir staining pattern within its glomeruli, while the staining against synapsin reveals a substructured organization in some glomeruli (Fig 2L, arrowhead).

Furthermore, well-defined TKRP-ir substructures were observed in the Dermestidae *D*. *maculatus* (Fig 2M) and in a bolboceratid species (Fig 2N), while in comparison, the Lucanidae (stag beetles) *L*. *cervus* (Fig 2O arrowheads) exhibited only weakly demarcated TKRP-ir substructures. In the Geotrupidae (earth-boring dung beetles) *G*. *stercorarius*, many of the glomeruli show weakly labeled TKRP-ir substructures (Fig 2P arrowheads), while some of the glomeruli are homogeneously labeled (Fig 2P arrow).

In all other species, inspection of TKRP-immunoreactivity of the ALs revealed a homogeneous (like for the Tenebrionidae (darkling beetles) *Tenebrio molitor* [Fig 2Q]) or evenly granular staining of the glomeruli (like in the Ptinidae (Spider beetles) *S*. *paniceum* [Fig 2R] or *O*. *funesta* [Fig 2L]), or no glomerular TKRP-immunoreactivity at all (like in the Lucanidae *P*. *muelleri* [Fig 2S]).

As already described for *O*. *funesta* (Fig 2L), in some of the examined species, the substructures are clearly labeled in stainings against synapsin and/or axonal actin (phalloidin), indicating dense synaptic networks. These species include a bolboceratid species that we could not further identify (Fig 2N arrowheads), *P*. *aemula* (Fig 2F arrowhead), *P*. *ephippiata* (Fig 2G arrowhead), *O*. *funesta* (Fig 2L arrowheads), *P*. *muelleri* (Fig 2S arrowhead) and the Scarabaeidae *Trichius spec*. (Fig 2T arrowheads), while some species showed indications for such substructures (as shown in digital supplement S1 Fig).

Comparable TKRP-ir substructures are unknown from other insects. TKRP-ir stainings in the AL of other insects have usually been described as homogeneous or uniform, like in *D*. *melanogaster* [33], *Spodoptera litura* [52], *Aedes aegypti* [35], *Periplaneta americana* [53], or *Leucophaea maderae* [54]. This also applies to insects with atypical glomeruli (like the many small microglomeruli in Acrididae [21,51]). For example, in the acridid *Schistocerca gregaria* TKRP-ir labeled fibers could be observed only within the interglomerular space [55].

### Innervation of the glomerular substructures

What types of TKRP-ir neurons contribute to the formation of the glomerular substructures in Coleoptera? In *A*. *tumida*, Kollmann et al. [40] could identify about 80 TKRP-ir LNs confined to the AL and entering the substructures of the glomeruli, but did not observe TKRP-ir fibers in the antennal nerve (AN) (excluding TKRP-ir OSNs) or TKRP-ir PNs or CNs. Similarly, in this work, all animals with TKRP-ir substructures show TKRP-ir LNs entering the AL glomeruli but a lack of TKRP-immunoreactivity in the AN and in the AL output tracts (PN axons). Also, no other TKRP-ir fibers possibly stemming from CNs had been observed.

**Fig 3:**
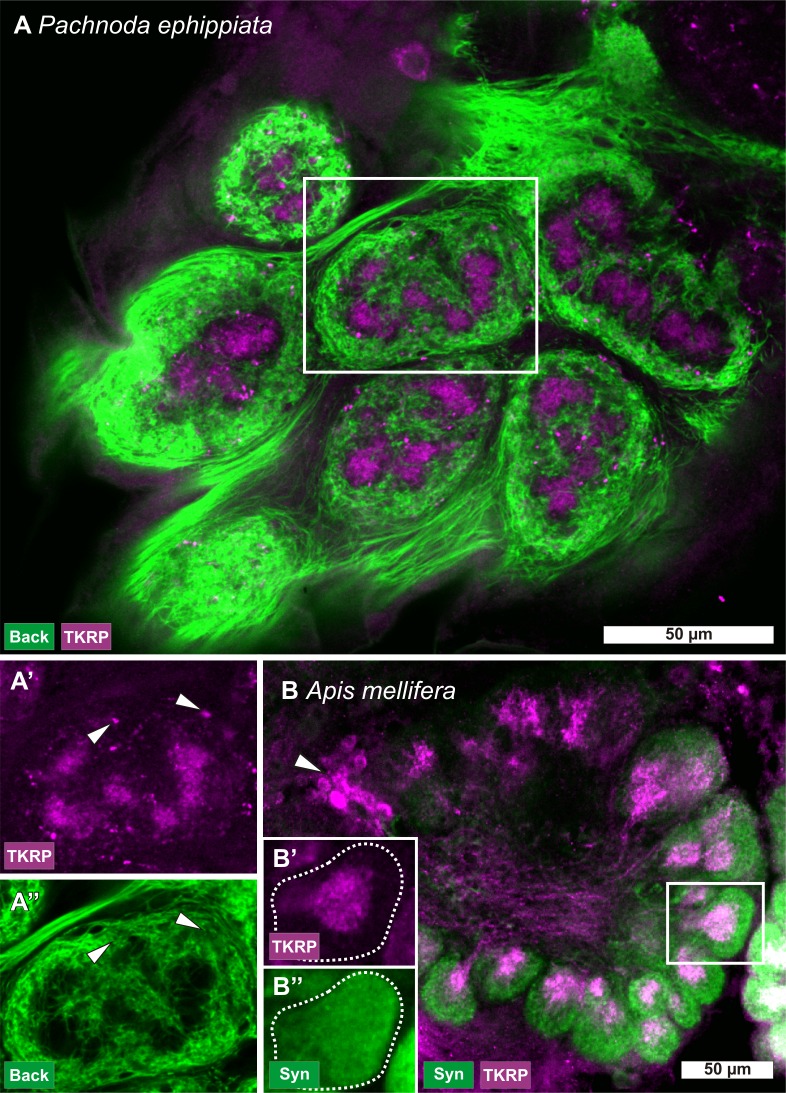
Antennal backfills and TKRP immunostaining. Antennal lobes (ALs) of *Pachnoda ephippiata* (Scarabaeidae) (A, A', and A'') and of *Apis mellifera* (B, B', and B''). Antennal backfills in *P*. *ephippiata* (A, A', A'') demonstrate that olfactory sensory neurons of the antenna (green) do not innervate the glomerular substructures, while the tachykinin related peptide immunoreactive (TKRP-ir) local neurons (LNs) (magenta) innervate mostly exclusive the glomerular substructures, save for several varicosities outside the substructures (A', A'' arrowheads). A' and A'' represent the labeling shown in the inset in A separated in the single channels. In *A*. *mellifera* (B, B', and B'') TKRP immunoreactivity in the glomerular core areas seems to stem primarily from LNs (magenta; arrowhead), while the whole glomeruli labeled with the synapsin antibody (Syn, green). B' and B'' represent the two separated labels shown in the inset in B.

To further elucidate a possible contribution of OSNs, we performed antennal backfills in a large scarabeid species, *P*. *ephippiata* and combined it with immunostainings against TKRP. The backfill stainings clearly leave out the spherical substructures (Fig 3A), showing that OSNs do not contribute to the innervation of the substructures. The TKRP immunostaining is mainly restricted to the substructures but several TKRP-ir varicosities occur in the remainder of the glomeruli (Fig 3A arrowheads).

In summary, we postulate that the glomerular substructures in beetles are innervated by TKPR-ir LNs but not TKRP-ir PNs, TKRP-ir CNs, or TKRP-ir OSNs. The glomerular substructures can typically be visualized via anti-synapsin immunostaining or phalloidin. The substructures may also be innervated by other non-TKRP-ir fibers stemming from LNs, PNs or CNs. That also other non TKRP-ir fibers may innervate the glomerular substructures is underlined by our finding in several beetle species where we found only the synapsin or phalloidin label without TKRP immunostaining. Deduced from the AN backfill experiment in *P*. *ephipiata*, we postulate that OSNs are in general not innervating the glomerular substructures in beetles.

## Discussion

Like the vertebrate central nervous systems, insect nervous systems are typically organized according to a basic bauplan. The bauplan of the central olfactory pathway of insects consists of the paired ALs, the first integration center for olfactory information and higher integration areas, including the MB and the LH [21,36]. The AL typically contain olfactory glomeruli that are usually interpreted as functional subunits for odor discrimination [56,57]. The principal glomerular organization can also be found in first order olfactory integration centers of other animal groups, including vertebrates [58,59], crustaceans [21], and mollusks [60,61].

The architecture of the insect AL has been studied in several species, ranging from basal species like e.g. silverfish to derived species like e.g. Drosophila (reviewed e.g. in [21]), but also in sister groups like e.g. Archaeognatha [62] or Collembola [63,64]. However, a systematic investigation including a higher number of specimen (particularly of one order) has so far been limited to the investigation of 37 species of Hawaiian Drosophila [65] and of 25 species of leaf-cutting ants [23]. Our study on 63 beetle species from 22 different families is the first study that allows a direct comparison within this largest insect group, the Coleoptera.

### Number of olfactory glomeruli covers a large range in Coleoptera

A comparison of glomerular numbers in 32 of the examined beetle species revealed a large variation. Glomerular numbers in the investigated beetle species ranged from 50–150 for regular glomeruli to about 1,000 microglomeruli in the examined ladybugs and diving beetles. In insects, the number of regular glomeruli ranging from about 40, like in drosophilids, up to 630 in the leaf-cutting ant *Apterostigma cf*. *mayri*. [23]. On the basis of available data, Schachtner et al. [21] speculated in their review that the number of regular glomeruli (excluding microglomeruli) in a given insect order might be well conserved and might exhibit only small variations reflecting specific ecological or ethological needs of the respective species. Meanwhile, not only our current findings in Coleoptera, but also data available from several studies in Hymenoptera that cover a range from 44 [66] up to 630 glomeruli [23] demonstrated that variations in glomeruli number within the same insect order can be quite large. The observed variation within the number of glomeruli in the Coleoptera may very likely results from their huge diversity and their many adaptations. However, in Coleoptera, at least at the family level, the number of glomeruli seems well conserved (Fig 1).

### Microglomeruli in particular Coleoptera families

Atypical ALs and glomeruli occur in various insects. For instance, the ALs of the Odonata *Libellula depressa* consist of small, spherical knots [67], while previously the Odonata ALs (like the ALs of Ephemeroptera) had been described as a- or nonglomerular [21,67]. In Hemiptera, the ALs have also been described as aglomerular (*Trioza apicalis* [68]) or as diminutive with only 13 glomeruli-like structures (*Scaphoideus titanus* [69]). Also the ALs of the Phthiraptera *Columbicola columbae* show no clearly defined glomeruli or any other compartments [70]. Conversely, as mentioned earlier, in Acrididae (like *Schistocerca gregaria* and *Chorthippus albomarginatus*), the ALs comprise thousands of small microglomeruli [21,51].

**Fig 4:**
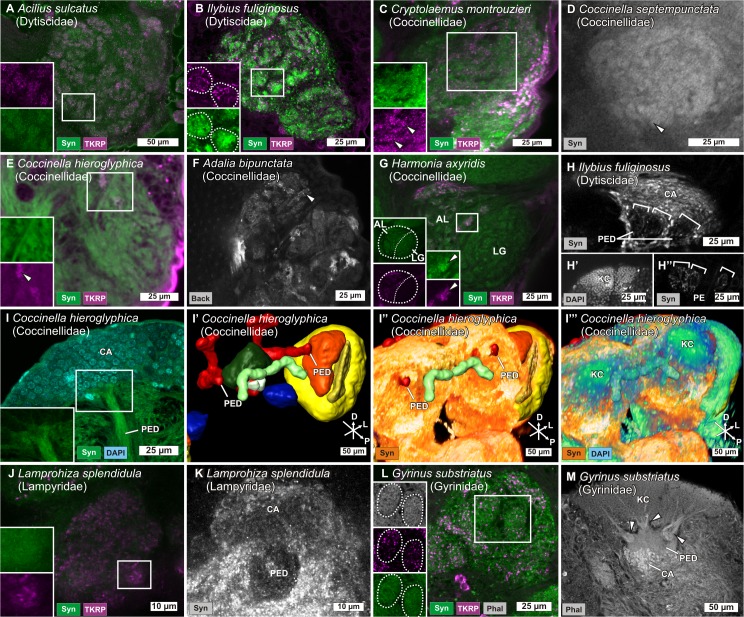
Antennal lobes and mushroom bodies of different Coleoptera. Antennal lobes (ALs) (A-G, J, L) and mushroom bodies (MBs) (H-I'', K, M) of different Coleoptera stained with antibodies against synapsin (Syn) and against tachykinin related peptides (TKRP) or labeled with DAPI or phalloidin (Phal). Boxes in the upper left show details of single glomeruli (A-C, E, G, J, L) or part of the MB (I) marked in the overview images, or they show the superstition between the AL and the lobus glomerulatus (LG) in the ladybug *Harmonia axyridis* (G). Arrowheads showing single glomeruli (C-G). Notice the trichotomy of the MB peduncle (PED) (square brackets in H and H'' and arrowheads in M) of the aquatic beetles *Ilybius fuliginosus* and *Gyrinus substriatus*. The calyx (CA) is absent in the ladybug *Coccinella hieroglyphica* as seen in the staining with Phal (I) and in the 3D-reconstruction and 3D-projektion (volume rendering) of Syn and DAPI (I'-I‴). In both cases, no calyx is visible between PED and Kenyon cells (KC). Orientation bars in I’ to I”‘: D = dorsal, P = posterior, L = lateral.

ALs with a microglomerular organization have already been observed in some beetle species. The ALs of diving beetles (Dytiscidae) have earlier been reported to show a nonglomerular organization or even to be totally absent in some representatives [41–43]. However, a recent in-depth study in ten representatives from this group found small and very numerous glomeruli in the ALs, similar to the microglomeruli of Acrididae [45]. This is in accordance with our own data from *A*. *sulcatus* with about 1,000 glomeruli per AL and *I*. *fuliginosus* with about 400–500 glomeruli per AL (Fig 4A and 4B).

Moreover, the current study revealed ALs with numerous small glomeruli, comparable to those observed in the diving beetles or Acrididae, in terrestrial Coccinellidae (ladybugs). Difficult to characterize in synapsin or phalloidin stainings, backfills and antibody stainings against TKRP helped to identify numerous small glomeruli (Fig 4C–4G arrowheads) and to differentiate the AL from a structure which we identified as the lobus glomerulatus (LG) (Fig 4G), a deutocerebral structure typically found in hemimetabolous insects but recently also reported to occur in beetles [39]. In hemimetabolous insects, the LG has been described to receive first order gustatory and mechanosensory input from the mouthparts [71]. A recent study in the beetle *Tribolium castaneum* suggested an innervation of the LG by OSNs based in sensilla of the mouthparts [39]. In all five investigated Coccinellidae, the ALs are remarkably small (Fig 1) and consist of numerous minute glomeruli (approximately 400–600 glomeruli per AL).

### Glomerular substructures in the Coleoptera

A recent study described a novel type of substructures in the olfactory glomeruli of the small hive beetle *A*. *tumida* that were evenly distributed across all glomeruli and innervated by TKRP-ir LN [40]. The glomerular substructures were also unmasked by anti-synapsin immunostaining that revealed a slightly higher synapsin density in the substructures compared to the surrounding glomerular neuropil [40]. The authors speculated that such a specialized organization may reflect a need to better handle the complex olfactory coding in a beehive in which these animals live as parasites. The current study shows that such an arrangement is by no means unique to *A*. *tumida*, as a similar organization of comparable TKRP-ir substructures was observed in 15 of the examined beetles. In addition, even more of the beetles showed substructures that were only revealed in synapsin and/or phalloidin labelings, but not evident solely based on TKRP immunostainings. These substructures are widely distributed across the phylogenetic tree but may be conserved within certain families.

### Phylogenetic distribution of substructured glomeruli in the Coleoptera

Substructures in olfactory glomeruli (TKRP-ir and non-TKRP-ir) occur in evolutionary distant families (Fig 1). In the 22 investigated families, TKRP-ir substructures occur in species of seven families. Adding glomerular substructures that were only revealed by synapsin/phalloidin labeling but showed no TKRP immunoreactivity, we found these structures in a total of 10 beetle families. Of the examined 15 species that belong to the superfamily of the Scarabaeoidea (comprising the four families Lucanidae, Bolboceratidae, Geotrupidae, and Scarabaeidae), ten species showed TKRP-ir substructures in all glomeruli, one shows TKRP-ir substructures in several glomeruli, and the remaining four species showed substructures visualized only in the synapsin/phalloidin labeling. We conclude that glomerular substructures are a conserved feature of the Scarabaeoidea. In the other examined polyphagous Coleoptera, the situation is less clear, either because only a single species of the respective family was studied or because we found species with and without clear substructures in the same family. For example, in the Tenebrionidae, half of the six examined species showed glomerular substructures either labeled with the anti-synapsin antibody or with phalloidin. A similar situation occurred in Cerambycidae with three of the five examined species displayed such substructures. In the silphids, we found one species (*N*. *vespilloides*) showing TKRP-ir substructures, while the other species (*P*. *atrata*) exhibits unstructured glomeruli. For Nitidulidae and Dermestidae, only one species was investigated, each showing the typical TKRP-ir glomerular substructures. All examined adephageous beetles lacked clear glomerular substructures. In summary, the spotty distribution of glomerular substructures across the different groups suggests that it is not a conserved feature in Coleoptera but may have evolved independently in several beetle taxa.

### Innervation of the glomerular substructures

To examine whether OSNs may in addition to the LNs contribute to the glomerular substructures, we exemplarily performed antennal backfills in a large scarabeid species, *P*. *ephippiata*, and combined it with immunostainings against TKRP. The results clearly underline the findings in *A*. *tumida* that OSNs do not contribute to the innervation of the substructures (Fig 3A) [40]. Based on these data, we propose that such glomerular substructures in beetles are generally organized according to this scheme. Further studies have to reveal whether other AL neuron types like PNs and CNs may also in addition contribute to the substructures. One such candidate that has been described in many insect species and that seems to be a basic feature of insect ALs is a paired serotonin-immunoreactive (5HT-ir) CN that typically innervates all olfactory glomeruli s [21,72]. In *A*. *tumida*, projections of the 5HT-ir CNs innervate all glomeruli but spare the substructures [40], suggesting that the projections of the 5HT-ir CNs are not part of the glomerular substructures of Coleoptera.

### How could glomerular substructures evolve from the basic non structured pattern?

Typically, insect OSNs expressing the same specific odorant receptor (OR) converge on the same glomerulus, with one OSN typically expressing only one specific OR [73–75]. In insects, an innervation of particular areas of a glomerulus by OSNs is known from several species including *D*. *melanogaster*, some lepidopteran species, and several Hymenoptera including some ant species, the hornet *Vespa velutina*, and the honeybee *A*. *mellifera* [21,30,76–86]. In these insects, the bulk of the glomeruli can be separated into two compartments: the outer cortex (also called cortex rind, cortex layer, cortical cap, cap, or peripheral area) and the inner core (sometimes termed base or basal area). OSN axons seem to project exclusively into the cortex [30,79,85,86]. Additionally, two types of LNs have been observed, one exclusively targeting the core region of a glomerulus and the other projecting into the core and the cortex [30,85,86]. With regards to PNs, uniglomerular PNs have branches in the core and cortex, multiglomerular PNs branch only in the cortex area [30,85,86]. Own data in *A*. *mellifera* showed TKRP-ir LNs innervating the core area (Fig 3B–3B'), comparable to immunostainings against the neuropeptide allatostatin [87]. However, multiple cores per glomerulus, like the multiple substructures in beetles have not been observed in *A*. *mellifera* (Fig 3B).

**Fig 5:**
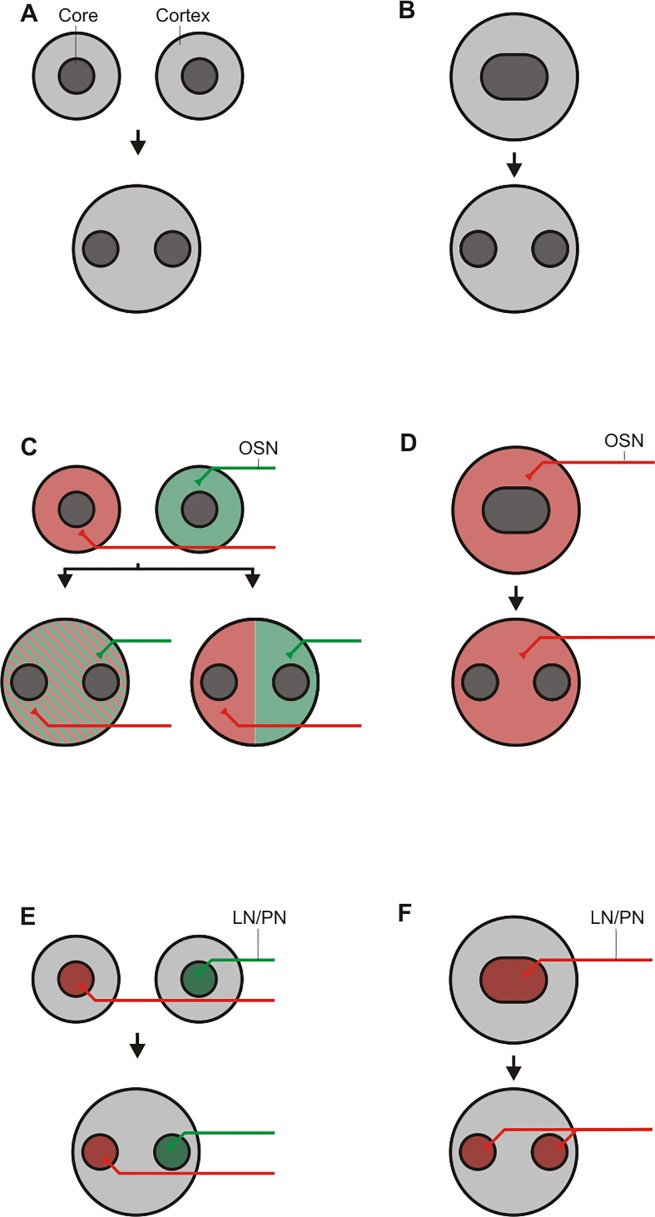
Considerations to the evolutionary origin the glomerular substructures. In principal, substructures could originate from the fusion of two (or more) glomeruli, each consisting of one cortex and one core, resulting in a glomerulus with one cortex and two cores / substructures (A). Substructures could also originate from a glomerulus with one cortex and one core and a subsequent division of the single core in multiple cores / substructures (B). C to F showing the possible principal innervation pattern as consequences of the two models (A and B) for olfactory sensory neurons (OSNs) (C and D) for local interneurons (LNs) respectively projection neurons (PNs) (E and F) (see text).

Assuming that a glomerulus with two compartments, as observed in *D*. *melanogaster*, Lepidoptera and Hymnoptera [21,30,76–86] reflects the basic architecture of a glomerulus of the holometabolous insects, multiple cores represent a derived situation. Glomeruli with multiple cores or substructures could be envisioned to have resulted from an incomplete fusion of such basic glomeruli, where the original core areas remained separated (Fig 5A). Alternatively, multiple glomerular substructures in a single glomerulus might have arisen through a differentiation of a single core into multiple cores (respectively substructures) (Fig 5B).

In the “fusion scenario” outlined above (Fig 5A), one would expect the fused glomerulus to inherit the innervation of its progenitors, i.e. to be innervated by OSNs carrying different specific ORs, either homogenously (Fig 5C left hand) or in separated regions (Fig 5C right hand). Alternatively, the “differentiation scenario” (Fig 5B), would suggest that the differentiated glomerulus should still be innervated by OSN expressing just one specific OR (Fig 5D). Future experiments utilizing transgenic lines and fluorescence *in situ* hybridization to label specific ORs, could help to answer this question by visualizing OSN innervation pattern of individual glomeruli. Furthermore, functional experiments using Calcium imaging may help to answer this question. In case only subareas of a given glomerulus would respond to different odorants, this would support the “fusion scenario”, while an overall response would support the “differentiation scenario”.

In addition, selective labeling of single uniglomerular LNs / PNs by dye filling with glass micropipettes would give insight whether the single substructures of one glomerulus are innervated via the same or different LN and would therefore help to understand how the multi cored glomeruli may have evolved. If a dye filled uniglomerular LN / PN projects only in one core of a glomerulus with multiple cores (Fig 5E), it is very likely, that this glomerulus originated from the fusion of single glomeruli. On the other hand, if a labeled uniglomerular LN / PN projects into all cores of a glomerulus (Fig 5F), this would support the idea, that the multiple substructures of a glomerulus result from a single glomerulus whose core has differentiated into multiple cores (respectively substructures).

### Multiple substructures in olfactory glomeruli outside Coleoptera

**Fig 6:**
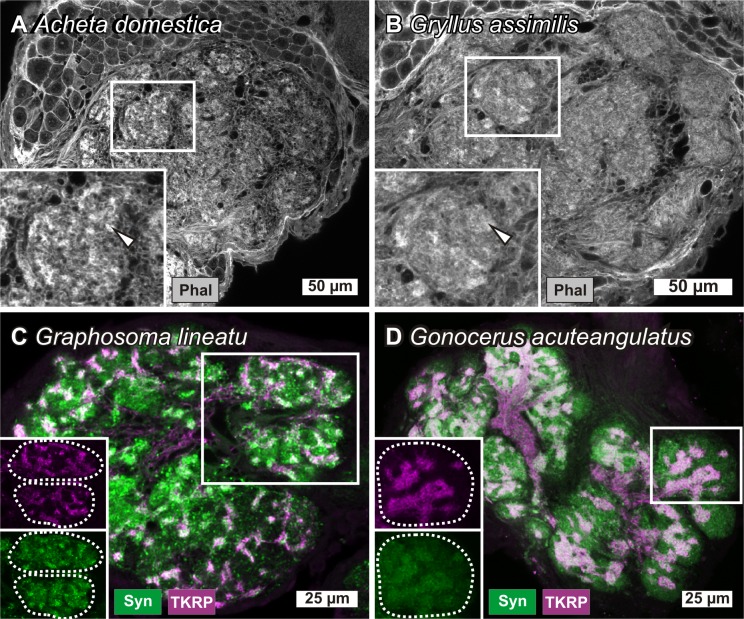
Glomerular substructures of hemimetabolous insects. Two Gryllidae *Acheta domestica* (A) and *Gryllusassimilis* (B) and two Hemiptera *Graphosoma lineatum* (C) and *Gonocerus acuteangulatus* (D). Boxes in the upper left of each image show a magnified view, respectively the two separated channels of the inset withinthe image. In *A*. *domestica* (A) and *G*. *assimilis* (B) *s*taining with phalloidin (Phal) revealed glomerular substructures (arrowheads), resembling the situation in *Gryllus bimaculatus* [51,88]. Staining with an antibody against tachykinin related peptide (TKRP) (magenta) and synapsin (Syn) (green) revealed irregular shaped and interconnected TKRP immunoreactive substructuring within the glomeruli of two Hemiptera species: *G*. *lineatum* (about 205 glomeruli) (C) and *G*. *acuteangulatus* (about 185 glomeruli) (D).

Up to now, glomeruli with clearly separated multiple substructures have been observed outside the beetles only once, i.e. in the Gryllidae *Gryllus bimaculatus*. Ignell et al. [51] and Yoritsune and Aonuma [88] described "microglomerular substructures" or "microglomerular clusters" within the "regular" glomeruli. Own stainings in the Gryllidae *Gryllus assimilis* and *Acheta domesticus* revealed also microglomerular substructures (Fig 6A and 6B arrowheads), showing that the observation in *G*. *bimaculatus* is not an isolated case. Both species lack anti-TKRP immunoreactivity within the entire ALs including the glomerular substructures. However, in contrast to our finding in the beetles, the glomerular substructures of *G*. *bimaculatus* are innervated by OSNs [20,51]. To explain the microglomeruli within the ALs of *G*. *bimaculatus*, Ignell et al. [51] argued, that glomeruli with restricted terminal arborizations of OSNs within one glomerulus can be found in many insect ALs (Diptera [89–91], Blattodea [92,93], Hymenoptera [94–97], Lepidoptera [79]). They hypothesized that such "multicompartmented uniquely identifiable glomeruli" could be fragmented into individual microglomeruli, potentially by a dichotomy of OSN axons before they enter a glomerulus. The microglomerular substructures observed in *G*. *bimaculatus* could thus be regarded as an evolutionary intermediate between "regular ALs with normal glomeruli" (known from most insects [21]) and microglomerular antennal lobes found in the Acrididae" [51]. However, microglomeruli contained within the glomeruli of Gryllidae are different to the beetle glomerular substrucutres as they are innervated by OSNs.

In two Hemiptera species, *Graphosoma lineatum* (Pentatomidae) and *Gonocerus acuteangulatus* (Coreidae) TKRP-immunostaining revealed approximately 200 glomeruli per AL (in each species) with a TKRP-ir staining pattern (Fig 6C and 6D) resembling the glomerular substructures found in Coleoptera. In contrast to the glomerular substructures observed in the Coleoptera, the TKRP-ir substructures of the two hemipteran species are of an irregular shape and are interconnected with each other (Fig 6C and 6D). Unlike in Gryllidae but similar to the TKRP-ir substructures of Coleoptera the TKRP-ir substructures of the Hemiptera are innervated by TKRP-ir LN, while the AN lacks any TKRP immunoreactivity. Whether the substructures of the two hemipteran species are more similar to the substructures of *G*. *bimaculatus*, which are innervated by OSNs [51,88], or whether they are more similar to the substructures of Coleoptera, which lack innervations by OSNs, remains to date unknown.

### Correlation of glomeruli architecture to brain architecture and lifestyle

On a gross ecological and ethological level (primarily terrestrial or aquatic habitat, nutrition; [98–102] substructured glomeruli in different Coleoptera could not be correlated with a specific lifestyle (Fig 1). There is also no correlation to the relative volumes of the four major brain neuropils (antennal lobes, optic lobes, central complex and mushroom bodies) (Fig 1) or to total / absolute volumes of the ALs.

Lifestyle (major nutrition) and the architecture of the ALs (size of AL or the number of its glomeruli) have also been found to be uncorrelated within Scarabaeidae [103]. However, Farris and Roberts [103] noted that differences in the feeding habits of Scarabaeidae (generalists vs. specialists) are reflected in the architecture of the MBs, rather than in the architecture / volume of the ALs. This might indicate that (at least in Coleoptera) lifestyle / preference of nutrition is rather reflected in the morphology of higher olfactory integration centers (the MBs), structures that are important for olfactory discrimination, learning, and memory storage and retrieval [57,104–106], than in the morphology of the primary olfactory integration centers (the ALs).

Numerous small glomeruli, comparable to the microglomeruli of Acrididae [21,51] could be identified in two coleopteran families, namely ladybugs and diving beetles. Despite obvious differences in habitat (terrestrial vs aquatic), both groups are primarily predatory and possess well-developed optic lobes with a large relative volume (Fig 1). A comparable microglomerular pattern can also be observed within the strongly visual orientating, predatory odonate *Libellula depressa* [67]. Interestingly, in all three taxa, the calyces show remarkable reductions or are even lacking (see below). Though not predatory, the locust *Schistocerca gregaria*, which also displays large optical neuropils, possesses ALs comprised of many microglomeruli [107]. While the correlation of numerous microglomeruli and large optical neuropils thus does not seem to necessarily imply predatory behavior per se, it points towards a possible and hitherto unstudied linkage between these two brain centers in distantly related insect taxa.

### Olfaction with atypical ALs

Besides regular-shaped AL glomeruli, as known from many insects like *D*. *melanogaster*, *A*. *mellifera*, and many moth species [21], several authors described insect ALs with deviating glomerular design, including ALs with numerous small glomeruli (microglomeruli), ALs with small and spherical knots, non-glomerular ALs or ALs comprising poorly demarcated and hardly distinguishable glomeruli (e.g. described as "ALs with glomerular-like structures") [21, 51,67–70]. In some cases, it has been hypothesized that such poorly developed ALs and/or the absence of clearly defined glomeruli might be an indicator of a poorly developed sense of smell or even anosmia. Odonata, for instance, have repeatedly been speculated to be (almost) anosmic [21, 41–43,108]. However, recent studies were able to confirm that the antenna of the odonate *L*. *depressa* possess about 120 OSNs in 40 sensilla [109] and that the odonate *Ischnura elegans* clearly responds to odors (in behavioral and electrophysiological assays [110]). Similarly, in *C*. *columbae* (a louse species exhibiting non-glomerular ALs), olfactory sensilla [111] and an odor response could be observed [112]. Aquatic beetles, often discussed to be anosmic [41–43], have also been demonstrated to respond to olfactory stimuli [113–115]. The notion that well-defined ALs with distinctive glomerular organization are not a mandatory prerequisite per se for olfaction is also underscored by our observations of microglomeruli within five ladybug species, which clearly possess antenna bearing olfactory sensilla [116–119] and which have repeatedly been demonstrated to respond to olfactory stimuli [120–125]. This is also in line with similar observations described for *S*. *gregaria* [126–128].

Insects with poorly developed ALs / glomeruli typically also exhibit poorly developed or even lacking MB calyces, as has been reported e.g. in Dytiscidae [43,45] (also confirmed by own observations [Fig 4H, 4H' and 4H'']), Odonata [42,43,67], and Hemiptera [42,68,69,129]. The five ladybug species investigated in this work also show small AL with diffusely demarcated microglomeruli (Fig 4C–4G) and also completely lack calyces, while the peduncles and the Kenyon cells are still clearly identifiable (Fig 4I–4I‴). The co-occurrence of small / lacking ALs or poorly defined olfactory glomeruli and small / lacking calyces seems to be a repeating pattern within insect neuroanatomy.

The firefly *L*. *splendidula*, which spends up to three years as a nymph that feeds on snails, before it transforms into the reproducing adult that lives for just about one week and does not feed [100,130], has only small ALs associated with equally small calyces (Figs 1, 4J and 4K). ALs and glomeruli in *L*. *splendidula* are unidentifiable based on stainings with a synapsin antibody or with phalloidin, and become barely visible in stainings with a TKRP antibody (Fig 4J). The small ALs with elusive glomerular boundaries and small calyces possibly reflect a reduced need for olfaction in the adult animals, which do not feed during their short life span (it even lacks developed mouthparts) and that find their mating partners primarily by visual cues [131,132]. Similar observations are known from the heteropteran *Diceroprocta semicincta*, which lives up to 17 years underground as feeding nymph before emerging as non-feeding, reproducing adult. In this short time period, the animals mainly focus on finding mating partners, using auditory stridulation cues rather than olfactory cues, which is reflected in a reduction of ALs and calyces [43,133].

Feeding habits have been speculated to be another reason for reduced or underdeveloped AL glomeruli. In Hemiptera, *S*. *titanus* is (at least in Europe) considered to be a feeding specialist that is monophagous on grapevine, while its relative *Hyalesthes obsoletus* is characterized as a generalist that feeds on different wild host plants. Notably, specialist *S*. *titanus* has approximately 150 times less OSNs than *H*. *obsoletus* and about 10 times less and more poorly defined glomeruli than *H*. *obsoletus* [69], with both species lacking calyces.

A contrary example, however, is provided by the whirligig beetle *Dineutus sublineatus*, which lacks ALs but has clearly identifiable calyces [44]. Previously, lacking ALs in aquatic beetles have been interpreted as a secondary loss. This statement is based on the fact that for land living animals, which re-adapt to an aquatic habitat, olfactory perception under water is very difficult, in consequence leading to a loss-of-function and (almost) anosmic animals [41,43,44]. The well-developed calyces in *D*. *sublineatus* had been explained by the strong involvement of the calyces in visual data processing. However, our data clearly show AL glomeruli (Fig 4L) and calyces (Fig 4M) within the whirligig beetle *G*. *substriatus*, questioning the general statement that whirligig beetles (like all other aquatic insects) lack ALs [44].

Finally, it must be emphasized that small, less developed, or lacking ALs (and in most cases the correlating small or lacking calyces) are most likely not an intrinsic feature of a given taxon (homology), but convergent adaptations to a similar (or even particular) lifestyle and specific ecological and ethological requirements.

## Materials and Methods

### Animals

Collection permits for protected insects according to "§ 45 Abs. 7 Nr. 3 des Gesetzes zur Neuregelung des Rechts des Naturschutzes und der Landschaftspflege (Bundesnaturschutzgesetz -BNatSchG) vom 29.07.2009 (BGBI. I, Nr. 51, S. 2542 ff)”by the local nature conservation authority Marburg (untere Naturschutzbehörde Marburg; 67 22 04) dated 2013.06.19, 2014.07.15, and 2014.08.07.

Three coccinellid species (*Adalia bipunctata*, *Cryptolaemus montrouzieri*, and *Coccinella septempunctata*) were purchased from "SAUTTER & STEPPER GmbH" (Ammerbuch, Germany). Three Tenebrionide species (*Alphitobius diaperinus*, *Zophobas morio*, and *Tenebrio molitor*) were obtained from "b.t.b.e. Insektenzucht GmbH" (Schnürpflingen, Germany). Two scarabaeid species, *Eudicella schultzeorum* and *Pachnoda aemula*, were acquired in November 2015 at the traditional “International Insect Exchange Fair” in Frankfurt am Main (Germany) and that is yearly carried out by the entomological association Apollo e.V. (see http://www.apollo-frankfurt.de/en/events/index.html; for regulations concerning the protection of species refer to http://www.apollo-frankfurt.de/download/BO2016en.pdf), six other species (*Chlorocala africana africana*, *Dicronorhina derbyana derbyana*, *Eudicella hornimanni*, and *Eudicella aethiopica*) were a generous gift from Jutta Renda from "Käferzucht" (Sinsheim-Hilsbach, Germany), and the Scarabaeidae *Pachnoda ephippiata* were a kind gift from Florian Schlusche (University of Konstanz, Germany). The Lucanidae *Phalacrognathus muelleri* and *Homoderus gladiator* were provided from a private rearing by Stefan Dippel (Momberg, Germany). Bolboceratidae specimens were a generous gift from Reinhard Predel and Susanne Neupert (University of Cologne, Germany) and were originally collected at Aha Hills, Namibia. Specimens of *Nicrophorus vespilloides* were a generous gift of Sandra Steiger (University of Ulm, Germany). *Dermestes maculatus* was kindly provided by Christian von Hoermann (University of Ulm, Germany). *Stegobium paniceum* and *Palembus dermestoide* were a generous gift from Mathias Schott (University of Gießen, Germany). The chrysomelid *Macroplea mutica* was a gift from Gregor Kölsch (University of Hamburg, Germany). The chrysomelid *Leptinotarsa decemlineata* and the curculionid *Gonipterus scutellatus* were kindly provided by Stefan Schütz (University of Göttingen, Germany). The following animals were collected in the vicinity of the Philipps University of Marburg (Germany), endangered animals we collected and dissected under permission from the conservation agency Marburg (Untere Naturschutzbehörde Marburg; 67 22 04—zim from 2013.06.19, 2014.07.15, and 2014.08.07): *Pyrochroa coccinea*, *Lagria spec*., *Coccinella hieroglyphica*, *Harmonia axyridis*, *Donacia versicolorea*, *Chrysolina sturmi*, *Lilioceris lilii*, *Rhagium bifasciatum*, *Molorchus minor*, *Pachytodes cerambyciformis*, *Alosterna tabacicolor*, *Stenurella melanura*, *Curculionidae spec*, *Hadroplontus litura*, *Liophloeus tessulatus*, *Otiorhynchus spec*., *Polydrusus pterygomalis*, *Lamprohiza splendidula*, *Rhagonycha fulva*, *Cantharis fusca*, *Ampedus sanguinus*, *Phosphuga atrata*, *Lucanus cervus*, *Amphimallon solstitiale*, *Oxythyrea funesta*, *Acilius sulcatus*, *Gyrinus substriatus*, *Pterostichus niger*, *Abax parallelepipedus*, *Calathus erratus*, *Coccinella hieroglyphica*, *Cicindela campestris*, *Hylecoetus dermestoides*, *Geotrupes stercorarius*, *Carabus nemoralis*. Data from the Nitidulidae *Aethina tumida* are obtained from Kollmann et al. [40]. Data for the Tenebrionid *T*. *castaneum* are obtained from Dreyer et al. [38], Binzer et al. [34], and Dippel et al. [39].

The foragers of *Apis mellifera* were kindly provided by the Bieneninstitut Kirchhain (Germany). Two Gryllidae (*Gryllus assimilis* and *Acheta domestica*) have been obtained from b.t.b.e. Insektenzucht GmbH. The two Heteroptera (*Gonocerus acuteangulatus* and *Graphosoma lineatum*) were collected close to the Philipps University of Marburg.

As the majority of animals were not reared under controlled conditions or were even collected from the wild, information on the exact age of the investigated specimens cannot be provided. Similarly, since a reliable determination of the sex of every specimen collected for this study proved infeasible, the sex of the animals was not taken into account in the present study. While we thus cannot rule out variations in AL architecture between different sexes of a given species, we did not encounter distinctive macroglomerular structures in the animals which glomeruli we counted.

### Phylogenetic relationships of the investigated animals

For Coleopteran gross phylogeny, we referred to Hunt et al. [8], who inferred phylogenetic relationships within the order based on sequence analyses of 18S rRNA, mitochondrial 16S rRNA and *cox1*.

For higher resolution of individual branches, we drew on the coccinellid phylogeny published by Magro et al. [134], the carabid phylogenies put forward by Maddison et al. [135] and Raupach et al. [136], the chryosmelid phylogeny provided by Gómez-Zurita et al. [137], and the phylogenetic trees for the superfamily Scarabaeoidea detailed in Browne and Scholtz [138,139] and Ahrens et al [140].

### Primary antisera

Similar to other insect studies [e.g. 34, 141, 142], a monoclonal primary antibody from mouse against a fusion protein consisting of a glutathione-S-transferase and the first amino acids of the presynaptic vesicle protein synapsin I coded by its 5′-end (SYNORF1; 3C11, #151101) was used to selectively label neuropil areas. The synapsin antibody was kindly provided by Dr. Erich Buchner (University of Würzburg, Germany) and was first described by Klagges et al. [143]. The antibody was used at a dilution of 1:100. The specificity of this antibody in the beetle *T*. *castaneum* has been demonstrated by Utz et al. [141].

The polyclonal antiserum against tachykinin-related peptide (TKRP) is against the *Locusta migratoria* tachykinin II (Lom-TK II, APLSGFYGVRamide) and was raised in rabbit. It was kindly provided by Dr. H. Agricola (K1-50820091) (University of Jena, Germany) and first described by Veenstra et al. [144]. In beetles, specificity of the antibody was confirmed for *T*. *castaneum* [34]. It was used at a dilution of 1:2,000.

### Secondary antibodies

Goat anti-mouse antibodies conjugated to Cy5 (GAM-Cy5) and goat anti-rabbit antibodies conjugated to Cy3 or Cy5 (GAR-Cy3 / GAR-Cy5) were used as secondary antibodies (each 1:300; Jackson ImmunoResearch, Westgrove, PA, USA).

### Further markers

Alexa Fluor 488-coupled phalloidin (Molecular Probes, Eugene, OR, USA) was used to visualize axonal f-actin and thus to reveal whole brain anatomy. It was used at a dilution of 1:200. DAPI (4',6-diamidinophenyindole; Sigma Aldrich, Steinheim, Germany) was used as a nuclear marker to identify neuronal somata. It was used at a dilution of 1:20,000. Neurobiotin (Vector Laboratories, Burlingame, UK) was used for the antennal backfills in a 4% solution, diluted in 1 M KCl. It was visualized with Cy3 conjugated streptavidin (1/200; Dianova, Hamburg, Germany).

### Double immunostainings of whole mount preparations

Brains were dissected under PBS (phosphate buffered saline; 0.01 M; pH 7.4) and were fixed overnight at 4°C in 4% PFA (paraformaldehyde; Roth, Karlsruhe, Germany) in PBS. In some cases, brains were transferred in PBS and were stored for several days at 4°C. Subsequently brains were washed 2–3 x 10–15 min (depending on the size of the brain), treated with collagenase-dispase (1 mg/ml in PBS; Sigma Aldrich) for 30–90 sec and washed 3–4 x 10–15 min. Afterwards brains were preincubated for 1 to 3 days in PBT (PBS added with 0.3% Triton-X 100, Sigma Aldrich) with 5% NGS (normal goat serum; Jackson Immuno Research) at 4°C. As primary antibodies we used anti-synapsin (1:100) in combination with anti-TKRP (1:20,000), diluted in PBT with 1% NGS. Brains were incubated for 2–5 days at 4°C. After rinsing (4–6 x 10–15 min) with PBT, brains were incubated in secondary antibodies (GAM-Cy5 and GAR-Cy3; 1:300; Jackson ImmunoResearch) and Alexa Fluor 488 Phalloidin (0.5%) and DAPI (1:20,000) in PBT with 1% NGS at 4°C for 2–5 days in the dark. After rinsing (4–6 x 10–15 min) with PBT, brains were dehydrated in an ascending alcohol series (30%, 50%, 70%, 90%, 95%, 2 x 100% ethanol, 3–7 min each) at room temperature. The tissue was then cleared to transparency in methyl salicylate (Merck, Darmstadt, Germany). Brains were finally mounted in resin (Permount, Fisher Scientific, Pittsburgh, PA, USA), using 2–10 layers of reinforcing rings as spacers (Zweckform, Oberlaindern, Germany) to prevent tissue compression.

### Backfills of the antenna

Cold-anesthetized animals were mounted with their backs on microscope slides, using dental wax (S-U-wax wire, 2.0 mm, hard; Schuler Dental, Ulm, Germany) and a soldering iron at low temperature (100°C; Solder-Unit ST 081; Star Tec Products, Bremen, Germany). The head was carefully waxed to the thorax and the base of the antenna was fixed with modeling clay (Das große Dino-Knet-Set; moses. Verlag GmbH, Kempen, Germany) and by using a soldering iron. The distal lamellate segments of the antenna were cut off. Glass micropipettes were drawn (Model P-97, Sutter Instrument, Novato, USA) from borosilicate glass (inner diameter, 0.75 mm; outer diameter, 1.5 mm; Hilgenberg, Malsfeld, Germany) and broken to a tip diameter matching the diameter of the antenna. Micropipettes were filled with 4% neurobiotin (Vector Laboratories, Burlingame, UK) solved in 1 M KCl and fitted onto the antenna stump. After 4 hours at RT micropipettes were removed, brains were dissected, fixed, digested with collagenase, and washed as described above. Brains were stained with an antibody against TKRP (1:20,000) and the marker Alexa Fluor 488 Phalloidin (0.5%) and DAPI (1:20,000) in PBT with 1% NGS for 3 (*H*. *axyridis*) or 5 (*P*. *ephippiata*) days at 4°C. Neurobiotin was visualized with Cy3-conjugated streptavidin (1:200; Dianova, Hamburg, Germany) and Lom-TK II was visualized with GAR-Cy5 (1:300) in PBT with 1% NGS for 2 (*H*. *axyridis*) or 4 (*P*. *ephippiata*) days at 4°C. Brains were embedded as described above.

### Data processing

Fluorescence was analyzed with a confocal laser scanning microscope (Leica TCS SP5, Bensheim, Germany). The following object lenses were used: 10x oil objective (HC PL APO CS 10x/0.40 IMM, working distance: 360 μm; Leica), 20x oil objective (HCX PL APO lambda blue 20x/0.70 Imm UV, working distance: 260 μm; Leica); 40x oil objective (HCX PL APO lambda blue 40x/1.25 Oil UV, working distance: 100 μm; Leica) und 63x glycerol objective (HCX PL APO 63x/1.30 Glyc 21°C CS working distance: 0.26 mm; Leica). Specimens were scanned with a resolution of 1024 x 1024 pixels, a line average of 2–3, speed of 200 Hz, a digital zoom of 1–3 and z-steps varying from 0.5 to 5 μm.

### Image segmentation, reconstruction, and visualization

Confocal image stacks were analyzed with AMIRA 5.2–5.6 (FEI, Hillsboro, OR, USA). For segmentation and reconstruction, we referred to Kurylas et al. [107]. In short, image stacks were edited in the "Segmentation Editor" of AMIRA. After labelling several sections in all three spatial directions (anterior to posterior, left to right and dorsal to ventral) of the neuropils / glomeruli, labeled segments were wrapped to gain a voxel-based 3-D model, which was then transformed (via “SurfaceGen”) into a polygonal surface model. A standard color code from Brandt et al. [145]. was used. Volume data was obtained using the function "MaterialStatistics", volume date for neuropils from *T*. *castaneum* and *Aethina tumida* were obtained from Dreyer et al. [38] and Kollmann et al. [40]. For image generation and final figure arrangements, snapshots were taken in AMIRA and subsequently processed by using global image adjustments (for example contrast and brightness optimization) in Corel Draw 13 (Corel Corporation, Ottawa, Ontario, CA).

### Determination of the number of glomeruli

To obtain the number of glomeruli for selected species, individual glomeruli within the ALs were reconstructed as described above. Due to the large amount of different species, only one AL per species was further investigated. Reconstructions were obtained from the most pronounced and well-defined labeling for each species (labeling with phalloidin, with an antibody against TKRP / synapsin, and/or backfills with neurobiotin). For many of the investigated species only few or often even one specimen had been available. For this reason, we used a standard staining protocol that was not optimized for each single species. The resulting staining quality of the ALs was typically sufficient to recognize the principal AL architecture but also often did not allow to reconstruct all individual glomeruli per AL. Therefore, and to accelerate the analysis to a reasonable time expense, we reconstructed only the glomeruli that were clearly distinguishable and calculated the total number from the average volume of the reconstructed glomeruli and the volume of the reconstructed overall neuropil volume of the respective AL (excluding the AL hub). From the 63 investigated species, we were able to seriously estimate the glomeruli number of 28 species. 5 of the 28 species possess numerous, small glomeruli, similar to the microglomeruli of Acrididae [21,51]. In case of microglomeruli we reconstructed 25 glomeruli before extrapolation of the total number of glomeruli. In the remaining 25 species, we reconstructed about 90% (11 species), 70 to 80% (6 species) or 30 to 70% (8 species) of the glomeruli before extrapolation. Data for from *T*. *castaneum* and *Aethina tumida* were obtained from Dreyer et al. [38], Kollmann et al. [40] and Dippel et al. [39].

## Supporting Information

S1 Fig Inhomogeneous staining in AL glomeruli. AL glomeruli of two beetle species from two families as examples for inhomogeneous staining that we interpret as indications for glomerular substructures (arrowheads) stained with phalloidin (Phal) and anti-synapsin antibody (Syn).
(TIF)

## Abbreviations

AL: antennal lobe
AN: antennal nerve
CN: centrifugal neuron
DAPI: 4 ', 6-diamidinophenyindoles
LN: Local interneuron
MB: mushroom body
OR: olfactory receptor
OSN: olfactory sensory neurons
PBS: phosphate buffered saline
PN: Projection neuron
TKRP: Tachykinin-related peptides
