# Supplementary figures and images for: Variations on a Theme: Antennal Lobe Architecture across Coleoptera
Source: PLoS One. 2016 Dec 14;11(12):e0166253. doi: 10.1371/journal.pone.0166253 (PMC5156346; doi:10.1371/journal.pone.0166253)

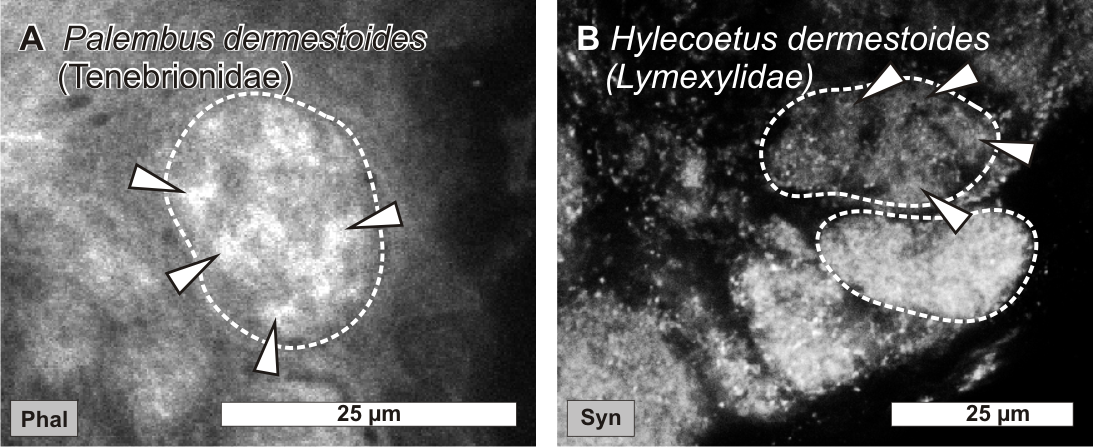

Supplement: S1 Fig — AL glomeruli of two beetle species from two families as examples for inhomogeneous staining that we interpret as indications for glomerular substructures (arrowheads) stained with phalloidin (Phal) and anti-synapsin antibody (Syn). (TIF) [file pone.0166253.s001.tif]
